# Nursing students’ experiences with using generative artificial intelligence: a descriptive qualitative study

**DOI:** 10.3389/fmed.2026.1916153

**Published:** 2026-09-30

**Authors:** Wenghao Wan, Xin Yu, Qianwen Guo, Zhengting Zhang, Miao Huang, Guohui Liu, Hengyu Zhou

**Affiliations:** 1 School of Nursing, Chongqing Medical University, Chongqing, China; 2 Department of Obstetrics, The Second Affiliated Hospital of Chongqing Medical University, Chongqing, China; 3 People’s Hospital Banan District, Chongqing, China

**Keywords:** generative artificial intelligence, nursing education, nursing students, qualitative study, student experiences

## Abstract

**Background:**

The rapid development of generative artificial intelligence (GenAI) has brought new opportunities to nursing education, while raising numerous concerns. Existing research has primarily focused on nursing students’ acceptance, attitudes, and willingness to use GenAI, whereas studies exploring their experiences and perceptions remain relatively limited.

**Objective:**

This study aimed to explore nursing students’ experiences with and perceptions of GenAI.

**Methods:**

A descriptive qualitative study was employed. Semi-structured interviews were conducted with full-time nursing students at Chongqing Medical University from March to April 2025 using purposive sampling, and the data were analyzed using conventional content analysis.

**Results:**

A total of 25 nursing students were interviewed, from which 3 themes and 10 subthemes were extracted: 1) perceptions and experiences of using GenAI (improving learning efficiency, concerns about information accuracy, concerns about overreliance on GenAI, and concerns about future career development); 2) factors influencing the experience of using GenAI (individual factors, information factors, and social environmental factors); and 3) expectations for integrating GenAI into nursing education (optimizing tool design, offering related courses, and promoting innovation in teaching methods).

**Conclusion:**

Nursing students in this study generally held positive but cautious attitudes toward GenAI. Although GenAI has gradually become an important support tool for learning and research among some nursing students, concerns regarding information accuracy, overreliance, and ethical and privacy risks warrant continued attention. Nursing educators should strengthen AI literacy education and guide students in using GenAI critically and responsibly. Meanwhile, efforts should be made to develop GenAI tools tailored to the needs of nursing education, strengthen education on ethical and privacy protection, and actively explore effective ways to integrate GenAI into nursing education.

## 1 Introduction

Nursing education plays a vital role in the healthcare field, aiming to cultivate nursing professionals with specialized knowledge, clinical skills, and humanistic care competencies to meet the growing demand for health services (1). In clinical practice, nurses assume a wide range of responsibilities, including patient assessment, health education, treatment administration, and emotional support. Therefore, integrating emerging technologies into nursing education is essential to ensure that nurses possess high-quality knowledge and skills (2).

In recent years, artificial intelligence (AI) has developed rapidly and has gradually been applied in fields such as medicine, finance, and journalism. Generative AI (GenAI) has attracted widespread attention due to its ability to generate text, images, and other content based on user input (3). Depending on the type of content generated, GenAI can be categorized into two types: text-to-text models (e.g., ChatGPT, DeepSeek) and text-to-image models (e.g., DALL-E, Midjourney) (4). Unlike traditional AI, which primarily classifies, predicts, or analyzes data, GenAI can generate new content based on user input and can also expand and create content based on text provided by the user. This “creativity” demonstrates that GenAI has the potential to advance nursing education (5).

The integration of AI into nursing education offers new solutions to traditional teaching methods, helping to improve students’ learning experiences, increase their learning motivation, and alleviate the faculty teaching burden (6, 7). As an emerging technology, GenAI can provide students with immediate feedback, support self-directed learning, facilitate clinical reasoning training, and offer personalized learning resources tailored to their learning needs (1, 8). Additionally, GenAI can assist with nursing academic writing, literature retrieval, and case analysis (9). By simulating real clinical scenarios, GenAI holds promise for providing nursing students with efficient and personalized learning experiences (8).

However, the application of GenAI in nursing education also faces several challenges. On the one hand, GenAI may generate inaccurate, misleading, or even fabricated information, which is commonly referred to as “AI hallucinations” (4, 10), thereby compromising the reliability of content. On the other hand, overreliance on GenAI may undermine students’ ability to think independently and critically, and raise concerns regarding academic integrity, data privacy, and ethics (11, 12). Furthermore, students’ insufficient understanding or negative attitudes toward GenAI may also hinder its application in nursing education (13). These challenges collectively influence students’ acceptance and effectiveness of using GenAI in actual teaching processes.

With the growing role of GenAI in healthcare, it is increasingly important to ensure that nursing students are adequately prepared and willing to use GenAI appropriately in their future learning and professional practice (13, 14). Research on nursing students’ use of GenAI has primarily employed quantitative approaches, such as questionnaire surveys, focusing on students’ acceptance, attitudes, behavioral intentions, and associated factors regarding GenAI use (13, 15, 16). These studies provide an overview of students’ attitudes toward GenAI, levels of acceptance, and tendencies to use it at the aggregate level. However, the reliance on standardized measurement instruments provides limited insight into how students understand and experience specific contexts of GenAI use and the complexity of their experiences.

Qualitative research can draw on participants’ lived experiences to provide deeper insights into the contextualized experiences and perceptions associated with GenAI use (17). Although some qualitative studies have begun to explore nursing students’ experiences with GenAI, existing research has largely focused on specific GenAI tools or specific tasks, such as the use of ChatGPT for academic writing (18) and assessment-related tasks (19). Other studies have focused on particular stages of nursing education, including first-year nursing students, nursing graduates, and postgraduate nursing students (19–21). These studies have enriched our understanding of nursing students’ experiences with GenAI; however, differences in the tools used, educational contexts, and participant populations make it difficult to fully capture nursing students’ experiences and perceptions of using GenAI in their routine learning and research activities. Therefore, this study employed a descriptive qualitative approach to explore the experiences and perceptions of Chinese nursing students using GenAI.

## 2 Methods

### 2.1 Study design

This study employed a descriptive qualitative research design. This approach emphasizes direct description of participants’ real experiences and presents findings in a manner close to the original data (22), making it suitable for exploring nursing students’ experiences of using GenAI. This study was reported in strict accordance with the Consolidated Criteria for Reporting Qualitative Research (COREQ) checklist (23).

### 2.2 Setting

This study was conducted at Chongqing Medical University, a public medical university located in western China that offers nursing education programs at both undergraduate and graduate levels. During the study period, students were permitted to use GenAI tools independently according to their individual learning and research needs.

### 2.3 Participants

Purposive sampling was used to recruit full-time nursing students from Chongqing Medical University as interview participants. The inclusion criteria were: 1) had used or were currently using GenAI tools (e.g., DeepSeek, Doubao); 2) were able to communicate normally without visual, auditory, reading, or writing impairments; and 3) volunteered to participate. The exclusion criterion was withdrawal or suspension from school for any reason.

### 2.4 Data collection

Based on the study objective, a preliminary interview guide was developed through literature review and team discussions. Two experts in nursing education and nursing informatics were invited to review and revise the content. No predetermined theoretical framework was used to guide the development of the interview guide or the data analysis. Before the formal interviews, pilot interviews were conducted with two nursing students who met the inclusion criteria, and the interview guide was further refined based on the pilot results. The final interview guide is provided in the Supplementary Material.

Data were collected from March to April 2025. Before the interviews, the researchers provided participants with detailed information about the study objective, content, and recording requirements, and assured them that the interview data would be kept strictly confidential and used solely for this study. Formal interviews were conducted after obtaining written informed consent. The interviews were conducted in a quiet and private office setting without interruption. Semi-structured face-to-face interviews were conducted by two master's students in nursing. One researcher was responsible for asking questions and guiding the interview, while the other was responsible for observing and taking notes on site.

All interviews were conducted in Mandarin Chinese and were recorded, with an average duration of 33 min. During the interviews, the researchers asked follow-up questions and sought clarification in real time based on participants’ responses to obtain richer information. Data collection was primarily guided by data saturation, defined as the point at which additional interviews yielded no new codes, themes, or meaningful information (24). After the 22nd interview, two researchers involved in data collection and analysis jointly assessed the data and, following discussion with the research team, determined that saturation had likely been reached. Three additional interviews (P23–P25) were subsequently conducted to further confirm saturation. The additional data were independently coded and generated no new codes or themes, confirming that data saturation had been achieved. A total of 25 participants were ultimately included in the study.

### 2.5 Data analysis

Data were analyzed using conventional content analysis (25). Within 24 h after each interview, the two researchers who had conducted the interview repeatedly listened to the audio recordings and independently transcribed the content verbatim, removing all identifiable personal information. The transcribed texts were then imported into NVivo 15.0 software for data management and analysis.

The data analysis proceeded as follows. The researchers repeatedly read the interview transcripts and immersed themselves in the data to gain a comprehensive understanding of the interview material. Meaning units of the text were identified and coded. Codes with similar meanings were grouped and compared to develop categories, which were subsequently synthesized into themes and subthemes.

Data analysis was conducted independently by two researchers. After completing the initial coding independently, the two researchers compared their coding results. For discrepant codes, they revisited the relevant meaning units in the original interview transcripts and their contextual information and discussed the rationale underlying their respective coding decisions. If discrepancies remained unresolved, a postdoctoral researcher on the team served as a third reviewer to adjudicate the disagreements until consensus was reached. A detailed example of the coding process is provided in Supplementary Table 1.

The interviews were transcribed verbatim in Chinese by the two researchers involved in data collection. For reporting purposes, representative quotations were translated into English by the first author, who is bilingual and experienced in academic writing in both Chinese and English, and independently back-translated and checked by a second bilingual researcher who was not involved in the initial translation. Any discrepancies arising during the translation process were resolved through discussion between the two researchers to ensure that the English quotations accurately reflected the meaning of the original Chinese text. All quotations were reviewed to preserve participants’ intended meanings while maintaining readability in English.

### 2.6 Researcher preparation and trustworthiness

The two researchers had completed coursework in qualitative research methods during their master's degree programs and received standardized training, equipping them with the competence to conduct qualitative studies. Our nursing backgrounds and research experience with GenAI may have increased our attention to issues such as learning efficiency, information reliability, ethics, and data security. We retrospectively considered these potential positional influences. However, because no formal reflexive journal was maintained and no written presuppositions or assumptions were established before the study commenced, we could not determine the extent to which these perspectives may have influenced the interview questions or interpretation of the data.

This study followed the four criteria for qualitative research rigor proposed by Lincoln and Guba (26): credibility, dependability, confirmability, and transferability. Multiple strategies were employed to ensure methodological rigor throughout the study. Credibility was enhanced through member checking and peer debriefing among the research team. After the initial coding and development of themes were completed, each participant was provided with a summary of the interpretations derived from their individual interview and asked to review the summary to confirm whether it accurately reflected their experiences. Dependability was ensured through independent coding by two researchers, ongoing comparison of coding results, and review and discussion of discrepancies within the research team. Throughout the research process, the interview guide, coding records, and documentation of theme development were retained to establish a comprehensive audit trail and enhance the confirmability of the findings. Transferability was supported by providing detailed descriptions of the research context, participant characteristics, data collection, and data analysis procedures, enabling readers to assess the applicability of the findings to other similar contexts.

## 3 Results

A total of 25 nursing students participated in this study, including 12 undergraduates and 13 master's students. The average age of participants was 23.04 ± 3.24 years. Seven participants used more than two GenAI tools simultaneously to support their learning and research activities. The GenAI tools used by the participants included DeepSeek (84%), Doubao (48%), Kimi (32%), Wenxin Yiyan (24%), ChatGPT (12%), and Qwen (12%). Participant characteristics are summarized in Table 1. The themes, subthemes, descriptions, and representative quotes are summarized in Table 2.

**Table 1:** Characteristics of the participants (*n* = 25).

No.	Gender	Age (years)	Educational Background	AI Tools Used*	Duration of Use (months)	Weekly Usage Frequency (times)
P1	Male	19	Bachelor's Degree	DS; KM	12	6–9
P2	Female	19	Bachelor's Degree	DB; KM	7	3–5
P3	Female	20	Bachelor's Degree	DS; DB	24	6–9
P4	Female	20	Bachelor's Degree	DS	18	6–9
P5	Female	21	Bachelor's Degree	DS; DB	24	≥10
P6	Female	22	Bachelor's Degree	DS; KM	12	3–5
P7	Male	20	Bachelor's Degree	DS; QW	24	3–5
P8	Female	21	Bachelor's Degree	WY	2	0–2
P9	Female	22	Bachelor's Degree	DS; DB; WY	3	3–5
P10	Male	21	Bachelor's Degree	DS; QW	24	6–9
P11	Male	22	Bachelor's Degree	DS; DB	20	≥10
P12	Female	22	Bachelor's Degree	DS; DB	18	6–9
P13	Female	23	Master's Degree	DS; DB; KM	7	≥10
P14	Female	24	Master's Degree	DB; KM	10	≥10
P15	Female	23	Master's Degree	DS; ChatGPT	8	≥10
P16	Female	23	Master's Degree	DS; DB; ChatGPT	6–12	6–9
P17	Female	24	Master's Degree	DS; WY	24	3–5
P18	Male	25	Master's Degree	DS; ChatGPT	24	≥10
P19	Male	23	Master's Degree	DS; DB	12	≥10
P20	Female	34	Master's Degree	DS; DB; KM	10	≥10
P21	Female	23	Master's Degree	KM	12	3–5
P22	Female	25	Master's Degree	DS; DB; WY	36	≥10
P23	Female	25	Master's Degree	DS; WY; QW	30	3–5
P24	Female	25	Master's Degree	DS; KM	12	3–5
P25	Female	30	Master's Degree	DS; KM; WY	5	6–9

* DS, DeepSeek; DB, Doubao; KM, Kimi; WY, Wenxin Yiyan; ChatGPT, ChatGPT; QW, Qwen.

**Table 2:** Themes, subthemes, descriptions, and representative quotes.

Theme	Subtheme	Description	Representative quote
Theme 1: Perceptions and Experiences of Using GenAI	Improving Learning Efficiency	The majority of participants reported that GenAI helped them quickly complete tasks such as understanding the literature, organizing content, and refining text, thereby saving time and improving learning efficiency.	“Before submitting papers, I used it (GenAI) to adjust the word count, polish the language, and organize the logical flow, which greatly improved my overall efficiency.” (P25)
	Concerns About Information Accuracy	Some participants noted that GenAI sometimes generated references that could not be verified through authoritative databases, cited authentic references that did not support the claims being made, and occasionally provided inaccurate data.	“The data that GenAI can access are limited, and much of the information it provides is not very accurate. Most of the data still need to be verified by myself.” (P15)
	Concerns About Overreliance on GenAI	Most participants were concerned that frequent or excessive use of GenAI might reduce opportunities for independent thinking and completing tasks autonomously, potentially affecting their thinking and creativity.	“I used to write many things out carefully by myself. But after using AI, I can't help but ask it first whenever I need to write something. I feel like my written expression skills are slowly deteriorating.” (P2)
	Concerns About Future Career Development	Some participants worried that the rapid development of AI technology might affect the future careers.	“If AI becomes more mature in the future, many tasks that used to require manual work might be automated. This makes me somewhat worried about my future employment.” (P10)
Theme 2: Factors Influencing the Experience of Using GenAI	Individual Factors	Some participants believed that personal interest in new tools, attitudes toward GenAI, and perceptions of potential risks could influence their user experience.	“I have always been quite interested in these new tools. After it (GenAI) became a trending topic, I actively sought out information about it and started trying to use it.” (P1)
	Information Factors	Participants pointed out that GenAI-generated content was often overly general, sometimes lacked logical clarity, and was not sufficiently comprehensive.	“When I was writing grant proposals or research plans, I tried several AI tools. The content they provided was often quite similar, and there was not much truly valuable information.” (P23)
	Social Environmental Factors	Participants reported that social environmental factors, including media exposure, peer influence, institutional policies, and the accessibility of GenAI tools, influenced their use of GenAI.	“My senior classmates had explored these tools before and would recommend which ones were good to use. Our supervisor also recommended some to us.” (P17)
Theme 3: Expectations for Integrating GenAI into Nursing Education	Optimizing Tool Design	Some participants reported that general-purpose GenAI tools lacked nursing-specific knowledge and up-to-date information, and suggested developing GenAI tools specifically tailored to the nursing profession.	“Medical knowledge updates too quickly. General-purpose AI completely fails to keep up with this pace, so it is necessary to have a GenAI tool specifically designed for nursing.” (P6)
	Offering Related Courses	Participants reported that they lacked knowledge and skills in prompt design and hoped that universities could provide technical guidance, organize specialized lectures, or offer AI-related courses.	“I think nursing undergraduate education should incorporate AI training courses, with instruction provided by professional technical staff, covering the theoretical knowledge of GenAI, usage procedures, and offering guidance on how to use it better.” (P10)
	Promoting Innovation in Teaching Methods	Some participants reported that GenAI could support innovations in teaching methods by providing interactive learning opportunities, such as virtual patients and scenario-based simulations.	“When practicing medical history-taking, I ask DeepSeek to act as a patient and develop my communication skills through ongoing conversations.” (P3)

### 3.1 Theme 1: perceptions and experiences of using GenAI

#### 3.1.1 Improving learning efficiency

The majority of participants reported that GenAI helped them quickly complete tasks such as understanding the literature, organizing content, and refining text, thereby saving time and improving learning efficiency. Some participants also noted that GenAI had gradually become an important support tool for their learning and research activities.

“When I come across something difficult to understand while reading an article, I send the relevant paper to DeepSeek and ask it to help me organize the main points and provide an interpretation. I used to spend a long time searching through the literature myself, but now I can get a clearer understanding of the key ideas within minutes.” (P13)

“Before submitting papers, I used it (GenAI) to adjust the word count, polish the language, and organize the logical flow, which greatly improved my overall efficiency.” (P25)

“GenAI has reduced my academic workload and greatly improved my learning efficiency. It has become an indispensable support tool in my daily studies.” (P7)

#### 3.1.2 Concerns about information accuracy

Although participants recognized the benefits of GenAI, some remained concerned about the accuracy of its generated content. Participants in this study reported that GenAI sometimes generated references that could not be verified through authoritative databases or cited authentic publications that did not support the claims being made. In addition, some participants noted that the data provided by GenAI were occasionally inaccurate, underscoring the need to independently verify AI-generated content.

“Sometimes it provides me with references that I simply cannot find, while some references do exist, but after reading the original articles, I found that they did not actually support the claims it made.” (P21)

“The data that GenAI can access are limited, and much of the information it provides is not very accurate. Most of the data still need to be verified by myself.” (P15)

“I asked Doubao to list the PMIDs and DOIs for the references. They all looked quite complete, but when I later checked them on PubMed, I found that much of the information did not match at all.” (P16)

#### 3.1.3 Concerns about overreliance on GenAI

Most participants were concerned that frequent or excessive use of GenAI might reduce opportunities for independent thinking and completing tasks autonomously, potentially affecting their thinking and creativity.

“I used to write many things out carefully by myself. But after using AI, I can’t help but ask it first whenever I need to write something. I feel like my written expression skills are slowly deteriorating.” (P2)

“If you rely on it (GenAI) to think through everything, over time your mental agility and creativity may be affected.” (P7)

“I have become somewhat dependent on it. Whenever the teacher asks a question in class, my first reaction is to ask it.” (P3)

#### 3.1.4 Concerns about future career development

Some participants expressed concerns about future employment, noting that as AI technology continues to advance, some tasks and support roles traditionally performed by humans may be replaced by AI.

“If AI becomes more mature in the future, many tasks that used to require manual work might be automated. This makes me somewhat worried about my future employment.” (P10)

“Although nursing emphasizes human-to-human communication, I still worry that certain simple positions might be replaced by AI in the future.” (P19)

### 3.2 Theme 2: factors influencing the experience of using GenAI

#### 3.2.1 Individual factors

Participants recalled their initial experiences with GenAI. Some became interested in and tried using GenAI after encountering media reports, while others explored it out of curiosity. These experiences indicated that some participants remained open to new tools and were willing to actively explore and experiment with them.

“I have always been quite interested in these new tools. After it (GenAI) became a trending topic, I actively sought out information about it and started trying to use it.” (P1)

“At first, I tried it (GenAI) out of curiosity. Later, I found it was really useful, so I have continued using it ever since.” (P6)

In this study, more than half of the participants expressed positive attitudes toward GenAI and recognized its value in learning and research. At the same time, some participants acknowledged its value while also expressing concerns about its potential negative effects.

“It used to take me an hour to search for information, but now I can obtain a lot of information within seconds, so I think this kind of tool is really valuable.” (P2)

“The rise of these tools has indeed brought great convenience to our study. But on the other hand, they can also limit our thinking to some extent.” (P7)

In addition, some participants expressed concerns about potential data privacy and ethical risks associated with the use of GenAI.

“If I use vague or substitute language to have it (GenAI) revise my paper, it affects efficiency. But if I directly input real data, I worry that it might leak my research data to others in the same field.” (P15)

“Nowadays, many learning tasks can be completed with the help of AI. I feel that this makes it easy for people to overlook their own thinking process, and involves issues related to academic integrity.” (P5)

#### 3.2.2 Information factors

Participants in this study identified several limitations of the information generated by GenAI. These primarily included: (1) the output was often overly general, with limited practically useful information; (2) the output sometimes lacked logical clarity, with poor coherence between different parts of some responses; and (3) the output was not sufficiently comprehensive.

“When I was writing grant proposals or research plans, I tried several AI tools. The content they provided was often quite similar, and there was not much truly valuable information.” (P23)

“Sometimes the response from GenAI looks quite detailed, but when I look at it more closely, I find that the different parts do not connect very well. It repeats some points, and the overall logic is not particularly clear.” (P14)

“I asked ChatGPT to help me search for relevant literature, but the results were not comprehensive. It could usually find only one or two articles, and the results were not what I was looking for.” (P25)

#### 3.2.3 Social environmental factors

Some participants were initially introduced to and learned about GenAI through online channels, such as social media and WeChat public accounts (Tencent Holdings Ltd., Shenzhen, China). Others learned about GenAI tools through recommendations and feedback from classmates and teachers.

“I first came across DeepSeek because it was recommended on a WeChat official account, and later I found it quite practical.” (P22)

“My senior classmates had explored these tools before and would recommend which ones were good to use. Our supervisor also recommended some to us.” (P17)

In addition, participants in this study reported several limitations on the use of GenAI, including access barriers to some foreign GenAI tools, explicit regulations on GenAI use in certain institutions, and usage limits on advanced features of some tools.

“In mainland China, tools like ChatGPT require a VPN to access, and the network connection is often unstable.” (P6)

“Some universities prohibit students from using GenAI to write academic papers.” (P20)

“Kimi’s features for creating PowerPoint presentations, generating images, and uploading files are subject to usage limits in the free version.” (P13)

### 3.3 Theme 3: expectations for integrating GenAI into nursing education

#### 3.3.1 Optimizing tool design

Participants reported that general-purpose GenAI tools have limitations in meeting the specific needs of nursing and keeping medical knowledge up to date. Some participants hoped that these tools could be further optimized and that nursing-specific GenAI applications could be developed, with relevant medical knowledge updated in a timely manner.

“Nursing emphasizes whole-cycle care and human-centered care. Currently, many AI tools still cannot truly align with the specific needs of the nursing profession.” (P3)

“Medical knowledge updates too quickly. General-purpose AI completely fails to keep up with this pace, so it is necessary to have a GenAI tool specifically designed for nursing.” (P6)

“Sometimes when I ask about more specialized nursing questions, general-purpose AI does not seem to understand them well and tends to provide rather general answers. It might be better to have AI specifically designed for medicine or nursing.” (P9)

In addition, participants in this study noted that the lack of verifiable data sources could affect the credibility of AI-generated content. Several participants hoped that future GenAI tools could be integrated with authoritative databases and provide authentic references.

“Sometimes the content generated by GenAI looks very convincing, but you don't know where it comes from, and there's no way to verify it.” (P15)

“If GenAI could be connected to databases such as CNKI, Wanfang Data, or PubMed and provide authentic literature sources, the information would be more convincing.” (P16)

#### 3.3.2 Offering related courses

Most participants reported uncertainty about how to use GenAI effectively, particularly due to a lack of knowledge and skills related to prompt design. They hoped that their universities could provide relevant technical guidance, offer specialized lectures, or introduce AI-related courses to help them better understand and use GenAI.

“I think nursing undergraduate education should incorporate AI training courses, with instruction provided by professional technical staff, covering the theoretical knowledge of GenAI, usage procedures, and offering guidance on how to use it better.” (P10)

“The school could invite AI experts to give lectures on using GenAI, which would help us gain a deeper understanding and further explore its potential functions.” (P19)

#### 3.3.3 Promoting innovation in teaching methods

Some participants noted that GenAI could provide interactive learning opportunities through virtual patients (VPs) and scenario-based simulations, which may help develop communication skills and enhance engagement in the learning process.

“When practicing medical history-taking, I ask DeepSeek to act as a patient and develop my communication skills through ongoing conversations.” (P3)

“When I use Doubao for case scenario practice, it asks further questions based on my responses. This interactive method gives me a stronger sense of engagement than simply reading case materials.” (P11)

## 4 Discussion

This study employed a descriptive qualitative research design to explore the experiences and perceptions of Chinese nursing students regarding the use of GenAI in nursing education. Nursing students in this study generally held positive but cautious attitudes toward GenAI. On the one hand, participants recognized its value in improving learning efficiency and supporting research activities. On the other hand, some participants expressed concerns about information accuracy, AI tool overreliance, data privacy, and future career development. In addition, participants’ use of GenAI was influenced by individual, informational, and social environmental factors, and they expressed high expectations for the integration of GenAI into nursing education. Overall, these findings suggest that GenAI has gradually become an important support tool for learning and research among some nursing students; however, its effective integration into nursing education still faces several challenges.

In this study, participants reported that their use of GenAI primarily involved information retrieval, literature reading, and academic writing. Similarly, a survey of Korean nursing students found that they used ChatGPT for writing papers and completing coursework to enhance their learning experiences (27). Notably, in contrast to previous studies on nursing students that mainly focused on ChatGPT (7, 19), participants in this study preferred domestic GenAI tools such as DeepSeek, Kimi, and Doubao, which is consistent with the findings of Yuan et al. (28). This preference may be partly attributable to differences in the regulatory environment and tool accessibility in mainland China, where ChatGPT is currently not accessible through conventional channels (29).

Subsequently, this study drew on the Unified Theory of Acceptance and Use of Technology (UTAUT) as an interpretive lens to understand nursing students’ experiences with GenAI use. According to UTAUT, individuals’ technology use behavior is primarily influenced by performance expectancy, effort expectancy, social influence, and facilitating conditions (30). In this study, most participants perceived GenAI as improving learning efficiency and supporting information retrieval and academic writing, reflecting positive performance expectations toward GenAI. This may be related to the ability of GenAI to rapidly retrieve and organize information, thereby reducing the time required for information searching and text processing (28). In addition, support from teachers and classmates, as well as promotion through social media, influenced participants’ exposure to and use of GenAI to some extent, suggesting that social influence may play a role in the use of GenAI. Some participants also reported that access to GenAI was affected by geographic restrictions and institutional policies and expressed a need for technical guidance. These findings suggest that facilitating conditions for GenAI use could be further improved. Therefore, educational institutions should provide stable and accessible GenAI tools along with necessary technical support (31), and offer structured GenAI courses or training to enhance students’ knowledge and skills for effective use of GenAI (32).

Although participants recognized the benefits of GenAI, some remained concerned about the accuracy of AI-generated content and the risk of overreliance on GenAI. A survey conducted in Egypt found that medical students also expressed concerns about the reliability of information provided by ChatGPT and its potential misuse in medical education (33). In this study, participants reported that GenAI sometimes generated inaccurate information, fabricated answers or references, and even provided references that did not support the claims being made. This phenomenon may be associated with the problem of “AI hallucinations” in GenAI (10), which remains difficult to address at the foundation model level. Labrague et al. reported that insufficient knowledge may hinder students’ effective use of AI tools (34). Therefore, nursing educators should help students strengthen their GenAI literacy and critical thinking skills and encourage them to critically evaluate AI-generated content (35). In addition, this study found that some participants were concerned that long-term reliance on GenAI might weaken their independent thinking and innovation abilities. In response to these concerns, educators should reconsider existing teaching and assessment models and proactively design evaluation methods that cannot be replaced by AI, such as oral presentations, group discussions, reflective journals, and case study design (11, 36), in order to encourage students to think actively rather than passively receive information.

Participants in this study also expressed concerns about their future career development, perceiving that advances in AI technology might lead to the replacement of some jobs. In practice, nursing is highly dependent on interpersonal communication, emotional support, and clinical judgment, capabilities, which are likely to remain difficult for AI to fully replace in the foreseeable future (37, 38). This concern may be associated with participants’ understanding of the capabilities and limitations of GenAI. A previous study found that similar concerns about job displacement were also reported among researchers (4), highlighting the need to promote and disseminate knowledge about GenAI. With the advancement of intelligent healthcare, cultivating nursing professionals who are well-prepared for AI-enabled healthcare environments has become one of the important tasks of nursing education (39). Nursing education should address the potential impact of AI on future employment while also helping students develop the ability to work collaboratively with AI. This would enable students to use AI appropriately to improve efficiency while maintaining professional judgment and the capacity to provide human-centered care.

Participants in this study expressed considerable concern about data privacy and ethical issues related to the use of GenAI in clinical and educational settings. Nursing practice involves substantial patient health information, and privacy protection has always been a fundamental ethical principle in nursing. Previous research has indicated that integrating large language models into healthcare is feasible; however, its widespread application depends on properly addressing data privacy and ethical issues (40). Merely remaining vigilant about GenAI-generated information is insufficient (41); systematic regulation is necessary. In China, the Expert Consensus on Ethical Governance of Clinical Applications of Generative Medical Artificial Intelligence, published in 2025, provided guidance on ethical governance for the clinical application of AI (42). However, clear ethical guidance and education on ethical boundaries remain limited in the nursing field (28). Therefore, educators, nursing professionals, and experts in related fields should collaborate to develop targeted guidelines to promote the appropriate and responsible use of GenAI in nursing (36). At the same time, many scholars have called for educational institutions to incorporate GenAI ethics into AI education frameworks for nursing teaching and learning, with the aim of fostering students’ critical thinking and analytical skills (16, 43, 44). At the institutional level, relevant authorities should also progressively improve regulatory policies and guidelines governing the use of GenAI in education and healthcare.

Notably, participants expressed high expectations for the integration of GenAI into nursing education. Given the highly specialized and practice-oriented nature of nursing education, participants perceived that general-purpose GenAI tools may not yet adequately meet their learning needs in areas such as specialized nursing knowledge and humanistic care. Some participants hoped that GenAI tools tailored to the specific characteristics and needs of nursing could be developed in the future. In addition, because the training data for GenAI are largely derived from the internet, the specialization and timeliness of its knowledge may be subject to certain limitations. Therefore, the accuracy of AI-generated content should remain an important consideration when applying GenAI in nursing. Several participants hoped that future GenAI tools could be integrated with authoritative databases to enhance the verifiability of AI-generated content. Previous research has suggested that integrating specialized knowledge bases and continuous knowledge-update mechanisms into large language models can improve the accuracy and reliability of generated content (45). In addition, participants noted that GenAI could support innovations in teaching, particularly in areas such as VPs, scenario-based simulation, and clinical communication training. Nursing education has long faced challenges such as limited practical resources and insufficient clinical teaching opportunities, whereas GenAI-driven VPs can provide students with a more flexible and personalized practice environment (46). Therefore, future development of GenAI tools for nursing should take nursing students’ learning needs into account and actively explore the integration of GenAI with teaching methods such as VPs and scenario-based simulation to facilitate the appropriate integration of GenAI into nursing education.

## 5 Limitations

This study has several limitations. First, all participants were recruited from a single university in southwestern China; therefore, the findings may not fully reflect the experiences of nursing students in other regions or institutional contexts. Second, the study included only nursing students with prior experience using GenAI, which may have resulted in a sample that was more likely to comprise students with higher levels of technology engagement or more open attitudes toward GenAI, thereby limiting the diversity of perspectives represented in the findings. Third, the study was limited to nursing students. Future research could include other relevant stakeholders, such as nursing educators, clinical nurses, and educational administrators, to provide a more comprehensive understanding of experiences with GenAI in nursing education and clinical practice from multiple perspectives. Fourth, we did not maintain a formal reflexive journal or establish written presuppositions or assumptions before the study commenced, which limited the traceability and transparency of the reflexive decision-making process. Finally, interviews conducted at a single time point could not capture potential changes in nursing students’ perceptions and attitudes as GenAI continues to evolve. Future research could adopt a longitudinal qualitative design to examine the dynamic changes in nursing students’ perceptions, attitudes, and experiences regarding GenAI use over time.

## 6 Conclusion

This study explored nursing students’ experiences of using GenAI in nursing education. Nursing students in this study generally recognized the value of GenAI in improving learning efficiency and supporting research activities, while also expressing concerns about information accuracy, overreliance, data privacy, and future career development. GenAI has gradually become an important support tool in the learning process for some nursing students; however, its effective application still requires multifaceted support. Nursing educators should help students develop appropriate attitudes toward GenAI use, maintain critical thinking, and actively explore innovative approaches to integrating GenAI into nursing education. Future efforts should focus on developing and optimizing GenAI tools tailored to the needs of nursing education, while strengthening education on AI ethics and data security to promote the appropriate and responsible use of GenAI in nursing education.

## Data Availability

The raw data supporting the conclusions of this article will be made available by the authors, without undue reservation.
