# Supplementary material for: Nursing students’ experiences with using generative artificial intelligence: a descriptive qualitative study
Source: Front Med (Lausanne). 2026 Sep 30;13:1916153. doi: 10.3389/fmed.2026.1916153 (PMC13661420; doi:10.3389/fmed.2026.1916153)
Supplement: Supplementary file 1 [file Table1.docx]

Supplementary Material

**Outline of the semi-structured interview:**

1. Could you describe your understanding and awareness of GenAI?

2. In your academic or clinical activities, what are the primary tasks for which you use GenAI?

3. What specific challenges have you faced when using GenAI tools in these contexts?

4. What is your overall perspective regarding the integration of GenAI into nursing education?

5. What key considerations should be prioritized to ensure the responsible use of GenAI?

6. How do you envision GenAI transforming nursing education in the future?
