# Supplementary material for: Nursing students’ experiences with using generative artificial intelligence: a descriptive qualitative study
Source: Front Med (Lausanne). 2026 Sep 30;13:1916153. doi: 10.3389/fmed.2026.1916153 (PMC13661420; doi:10.3389/fmed.2026.1916153)
Supplement: Supplementary file 2 [file Table2.docx]

Supplementary Material

**Supplementary Table 1. Examples of the Coding Process**

| **Participant** | Raw quote | Initial code | Category | Subtheme |
| --- | --- | --- | --- | --- |
| P2 | “It used to take me an hour to search for information, but now I can obtain a lot of information within seconds, so I think this kind of tool is really valuable.” | Saving time on information retrieval | Saving time | Improving learning efficiency |
| P12 | “After translating a section of an English-language article, I ask Doubao to polish the Chinese translation so that it reads more naturally and fluently.” | Polishing translated texts | Language and text processing |  |
| P7 | “If I come across something I don’t understand, I just need to take a photo and upload it, and GenAI can help clarify it for me.” | Quickly addressing learning-related questions | Learning-related problem solving |  |
| P10 | “I used to spend half an hour organizing meeting notes, but now GenAI can generate a summary directly, reducing the amount of repetitive work.” | Reducing the time spent organizing notes | Saving time |  |
| P9 | “When I come across a concept I am unclear about while reviewing, I ask DeepSeek. It can quickly identify the relevant information and provide a clear explanation.” | Rapidly retrieving and explaining relevant information | Information acquisition and comprehension |  |
| P17 | “Before starting my thesis proposal, I was unsure about which direction to take, so I asked DeepSeek to suggest several potential research angles. After reviewing them, I suddenly had a much clearer idea of where to go.” | Providing research ideas | Supporting academic thinking |  |
| P13 | “When reading English-language literature, I often come across technical terms that I do not understand, so I copy the entire article into it (GenAI) and ask it to translate and explain it. This has greatly improved my reading efficiency.” | Translating and explaining specialized literature | Information acquisition and comprehension |  |
| P20 | “After completing the questionnaire survey, I uploaded all the data to AI and asked it to summarize the results in a table.” | Creating tables and processing data | Research task support |  |

Note: The table presents selected coding examples from the subtheme “Improving learning efficiency” and does not represent all coded data within this subtheme.
